# Supplementary material for: The Impact of Foehn Wind on Mental Distress among Patients in a Swiss Psychiatric Hospital
Source: Int J Environ Res Public Health. 2022 Aug 30;19(17):10831. doi: 10.3390/ijerph191710831 (PMC9518389; doi:10.3390/ijerph191710831)
Supplement: Supplementary file 1 [file ijerph-19-10831-s001.zip › Mikutta_Pervilhac_SuppTableS1.pdf]

Supplementary Table S1: ICD codes of the admission and discharge groups

ICD-10 Code	Admission		Discharge	
	N	%	N	%
F00.0	4	0.0%	2	0.0%
F00.1	1	0.0%	1	0.0%
F00.2	3	0.0%	2	0.0%
F00.9	6	0.1%	5	0.0%
F01.0	6	0.1%	4	0.0%
F01.1	3	0.0%	2	0.0%
F01.2	6	0.1%	5	0.0%
F01.3	12	0.1%	11	0.1%
F01.8	6	0.1%	5	0.0%
F01.9	16	0.2%	15	0.1%
F02.3	1	0.0%	1	0.0%
F02.8	1	0.0%	1	0.0%
F03	51	0.5%	46	0.4%
F05.0	8	0.1%	8	0.1%
F05.1	42	0.4%	41	0.4%
F05.8	14	0.1%	13	0.1%
F05.9	19	0.2%	19	0.2%
F06.2	14	0.1%	13	0.1%
F06.3	8	0.1%	8	0.1%
F06.7	6	0.1%	5	0.0%
F06.8	1	0.0%	1	0.0%
F06.9	18	0.2%	15	0.1%
F07.0	12	0.1%	12	0.1%
F07.2	2	0.0%	2	0.0%
F07.8	5	0.0%	5	0.0%
F07.9	4	0.0%	4	0.0%
F09	1	0.0%	1	0.0%
F10.0	80	0.8%	80	0.8%
F10.1	67	0.6%	66	0.6%
F10.2	1087	10.4%	1079	10.4%

F10.3	7	0.1%	7	0.1%
F10.4	3	0.0%	3	0.0%
F10.6	6	0.1%	6	0.1%
F10.7	2	0.0%	2	0.0%
F10.8	1	0.0%	1	0.0%
F11.0	2	0.0%	2	0.0%
F11.1	4	0.0%	4	0.0%
F11.2	86	0.8%	86	0.8%
F11.3	4	0.0%	4	0.0%
F11.5	3	0.0%	3	0.0%
F11.9	1	0.0%	1	0.0%
F12.0	1	0.0%	1	0.0%
F12.1	16	0.2%	16	0.2%
F12.2	76	0.7%	75	0.7%
F12.3	2	0.0%	2	0.0%
F12.5	9	0.1%	9	0.1%
F12.7	3	0.0%	3	0.0%
F13.0	3	0.0%	3	0.0%
F13.1	6	0.1%	6	0.1%
F13.2	110	1.1%	109	1.0%
F13.3	1	0.0%	1	0.0%
F13.4	1	0.0%	1	0.0%
F13.7	2	0.0%	2	0.0%
F14.0	3	0.0%	3	0.0%
F14.1	19	0.2%	19	0.2%
F14.2	70	0.7%	70	0.7%
F14.5	2	0.0%	2	0.0%
F15.0	1	0.0%	1	0.0%
F15.1	1	0.0%	1	0.0%
F15.2	17	0.2%	17	0.2%
F15.3	2	0.0%	2	0.0%
F15.7	1	0.0%	1	0.0%
F17.2	1	0.0%	1	0.0%

F19.0	4	0.0%	4	0.0%
F19.1	4	0.0%	4	0.0%
F19.2	92	0.9%	90	0.9%
F19.3	1	0.0%	1	0.0%
F19.5	3	0.0%	3	0.0%
F19.7	1	0.0%	1	0.0%
F20.0	439	4.2%	436	4.2%
F20.1	8	0.1%	8	0.1%
F20.2	26	0.2%	25	0.2%
F20.3	4	0.0%	4	0.0%
F20.4	1	0.0%	1	0.0%
F20.5	25	0.2%	25	0.2%
F20.6	2	0.0%	2	0.0%
F20.8	2	0.0%	2	0.0%
F20.9	5	0.0%	5	0.0%
F21	5	0.0%	5	0.0%
F22.0	34	0.3%	34	0.3%
F22.8	1	0.0%	1	0.0%
F22.9	2	0.0%	2	0.0%
F23.0	46	0.4%	46	0.4%
F23.1	21	0.2%	21	0.2%
F23.2	17	0.2%	17	0.2%
F23.3	8	0.1%	8	0.1%
F23.8	5	0.0%	5	0.0%
F23.9	12	0.1%	12	0.1%
F24	1	0.0%	1	0.0%
F25.0	46	0.4%	45	0.4%
F25.1	94	0.9%	93	0.9%
F25.2	40	0.4%	40	0.4%
F25.8	3	0.0%	3	0.0%
F25.9	11	0.1%	11	0.1%
F28	3	0.0%	3	0.0%
F29	5	0.0%	4	0.0%

F30.0	2	0.0%	2	0.0%
F30.1	3	0.0%	3	0.0%
F30.2	1	0.0%	1	0.0%
F30.8	1	0.0%	1	0.0%
F31.0	53	0.5%	53	0.5%
F31.1	43	0.4%	43	0.4%
F31.2	20	0.2%	20	0.2%
F31.3	120	1.1%	120	1.2%
F31.4	86	0.8%	86	0.8%
F31.5	11	0.1%	10	0.1%
F31.6	27	0.3%	27	0.3%
F31.7	3	0.0%	3	0.0%
F31.8	25	0.2%	25	0.2%
F31.9	7	0.1%	7	0.1%
F32.0	44	0.4%	44	0.4%
F32.1	1792	17.1%	1779	17.1%
F32.2	438	4.2%	437	4.2%
F32.3	54	0.5%	53	0.5%
F32.8	3	0.0%	3	0.0%
F32.9	12	0.1%	11	0.1%
F33.0	45	0.4%	45	0.4%
F33.1	2001	19.1%	1996	19.2%
F33.2	1210	11.6%	1200	11.6%
F33.3	128	1.2%	127	1.2%
F33.4	11	0.1%	11	0.1%
F33.9	2	0.0%	2	0.0%
F34.1	2	0.0%	2	0.0%
F34.8	2	0.0%	2	0.0%
F34.9	1	0.0%	1	0.0%
F38.0	1	0.0%	1	0.0%
F40.00	3	0.0%	3	0.0%
F40.01	11	0.1%	11	0.1%
F40.1	10	0.1%	10	0.1%

F40.2	1	0.0%	1	0.0%
F41.0	46	0.4%	46	0.4%
F41.1	62	0.6%	62	0.6%
F41.2	50	0.5%	49	0.5%
F41.3	7	0.1%	7	0.1%
F41.8	3	0.0%	3	0.0%
F41.9	3	0.0%	3	0.0%
F42.0	6	0.1%	6	0.1%
F42.1	9	0.1%	9	0.1%
F42.2	16	0.2%	16	0.2%
F42.9	2	0.0%	2	0.0%
F43.0	73	0.7%	72	0.7%
F43.1	193	1.8%	193	1.9%
F43.2	252	2.4%	250	2.4%
F43.8	7	0.1%	7	0.1%
F43.9	103	1.0%	103	1.0%
F44.0	2	0.0%	2	0.0%
F44.4	4	0.0%	4	0.0%
F44.5	4	0.0%	4	0.0%
F44.7	5	0.0%	5	0.0%
F44.81	30	0.3%	30	0.3%
F44.88	1	0.0%	1	0.0%
F44.9	36	0.3%	36	0.3%
F45.0	10	0.1%	10	0.1%
F45.1	4	0.0%	4	0.0%
F45.2	6	0.1%	6	0.1%
F45.3	1	0.0%	1	0.0%
F45.30	1	0.0%	1	0.0%
F45.37	1	0.0%	1	0.0%
F45.40	8	0.1%	8	0.1%
F45.41	7	0.1%	7	0.1%
F45.9	1	0.0%	1	0.0%
F48.0	1	0.0%	1	0.0%

F48.1	2	0.0%	2	0.0%
F48.8	1	0.0%	1	0.0%
F50.0	4	0.0%	3	0.0%
F50.00	1	0.0%	1	0.0%
F50.1	2	0.0%	2	0.0%
F50.2	3	0.0%	3	0.0%
F50.9	1	0.0%	1	0.0%
F51.0	1	0.0%	1	0.0%
F53.1	1	0.0%	1	0.0%
F60.0	5	0.0%	5	0.0%
F60.1	1	0.0%	1	0.0%
F60.2	1	0.0%	1	0.0%
F60.30	24	0.2%	24	0.2%
F60.31	136	1.3%	136	1.3%
F60.5	2	0.0%	2	0.0%
F60.6	6	0.1%	6	0.1%
F60.7	2	0.0%	2	0.0%
F60.8	5	0.0%	5	0.0%
F60.9	1	0.0%	1	0.0%
F61	24	0.2%	24	0.2%
F62.0	2	0.0%	2	0.0%
F62.80	2	0.0%	2	0.0%
F62.9	2	0.0%	2	0.0%
F63.0	9	0.1%	9	0.1%
F63.2	1	0.0%	1	0.0%
F63.8	5	0.0%	5	0.0%
F63.9	9	0.1%	9	0.1%
F64.0	1	0.0%	1	0.0%
F68.1	1	0.0%	1	0.0%
F69	2	0.0%	2	0.0%
F70.0	3	0.0%	3	0.0%
F70.1	8	0.1%	8	0.1%
F70.8	1	0.0%	1	0.0%

F71.1	10	0.1%	10	0.1%
F71.8	1	0.0%	1	0.0%
F72.1	1	0.0%	1	0.0%
F73.0	1	0.0%	1	0.0%
F73.1	1	0.0%	1	0.0%
F79.8	1	0.0%	1	0.0%
F79.9	1	0.0%	1	0.0%
F80.1	1	0.0%	1	0.0%
F84.1	1	0.0%	1	0.0%
F84.5	7	0.1%	7	0.1%
F90.0	38	0.4%	38	0.4%
F90.1	2	0.0%	2	0.0%
F91.0	1	0.0%	1	0.0%
F91.1	1	0.0%	1	0.0%
F95.2	1	0.0%	1	0.0%
F99	1	0.0%	1	0.0%
G12.2	2	0.0%	2	0.0%
G20.11	1	0.0%	1	0.0%
G20.90	1	0.0%	1	0.0%
G30.0	11	0.1%	11	0.1%
G30.1	23	0.2%	23	0.2%
G30.8	2	0.0%	2	0.0%
G30.9	9	0.1%	9	0.1%
G31.0	6	0.1%	6	0.1%
G35.0	1	0.0%	1	0.0%
G35.20	1	0.0%	1	0.0%
G93.3	1	0.0%	1	0.0%
Z73	1	0.0%	1	0.0%
Total	10465	100.0%	10384	100.0%
