# Supplementary figures and images for: The Impact of Foehn Wind on Mental Distress among Patients in a Swiss Psychiatric Hospital
Source: Int J Environ Res Public Health. 2022 Aug 30;19(17):10831. doi: 10.3390/ijerph191710831 (PMC9518389; doi:10.3390/ijerph191710831)

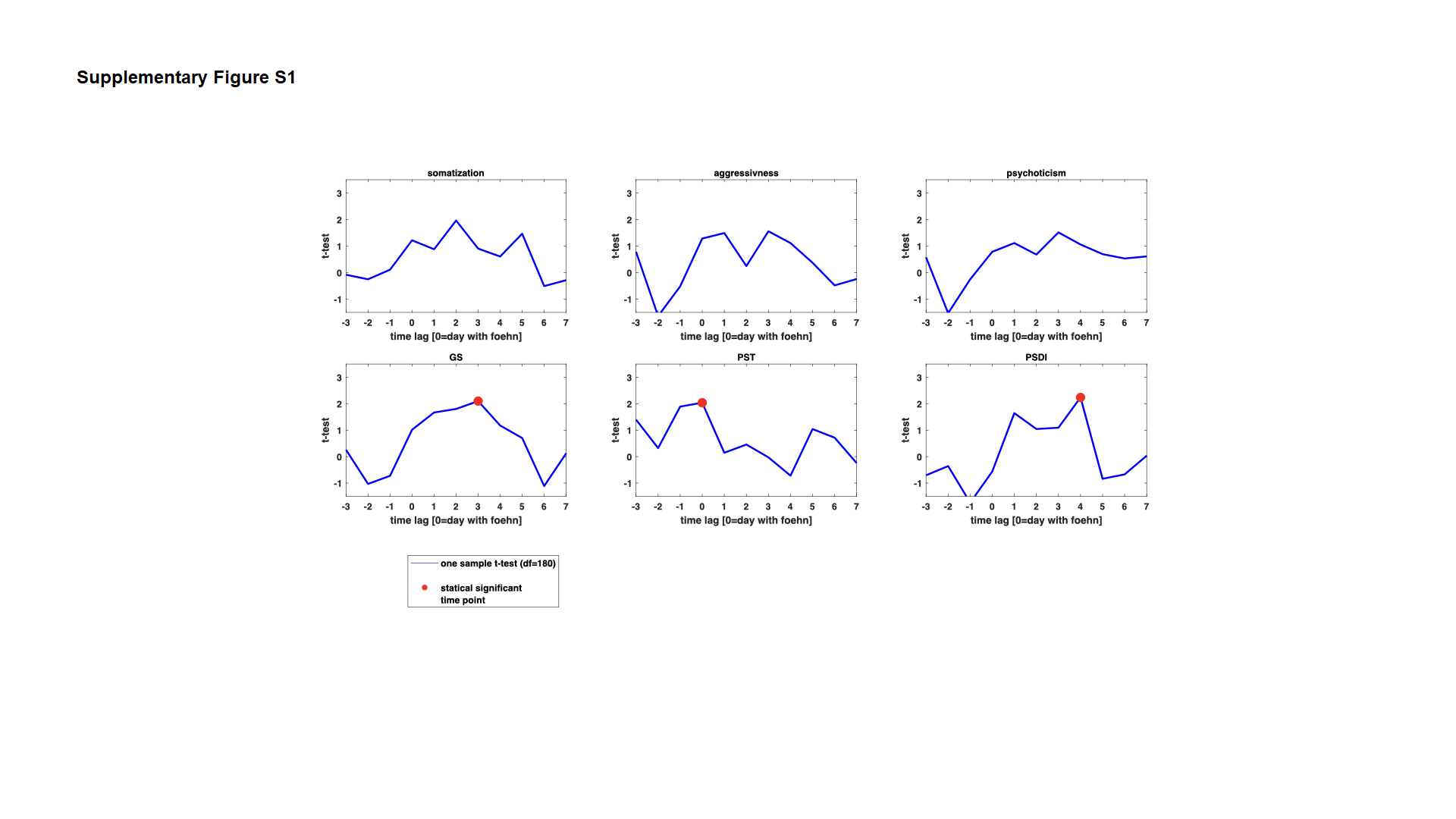

Supplement: Supplementary file 1 [file ijerph-19-10831-s001.zip › Mikutta_Pervilhac_Supp_Figures.S1.tiff]

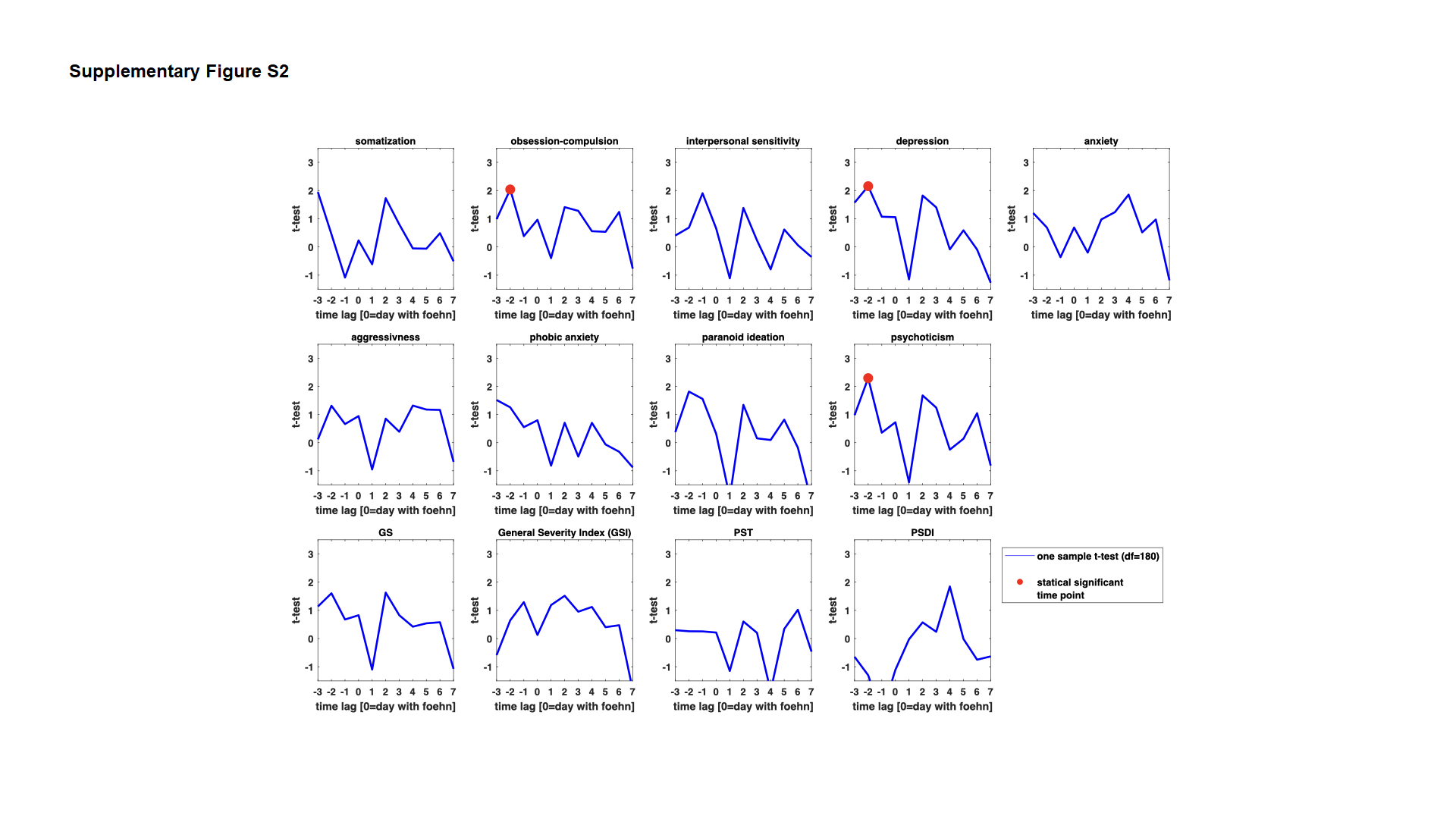

Supplement: Supplementary file 1 [file ijerph-19-10831-s001.zip › Mikutta_Pervilhac_Supp_Figures.S2.tiff]
